# Supplementary material for: Analysis of the utilization of traditional medicine in Korea over 10 years (2013–2022): A repeated cross-sectional study using national health insurance data
Source: PLoS One. 2025 Apr 8;20(4):e0321517. doi: 10.1371/journal.pone.0321517 (PMC11977961; doi:10.1371/journal.pone.0321517)
Supplement: S1 Table — (PDF) [file pone.0321517.s001.pdf]

S1 Table. Number of medical institutions, beds, and medical doctors in Korea between 2013 and 2022

Year	Institutions					Beds					WM Doctors				TKM Doctors			
	Total	WM Hospitals	WM Clinics	TKM Hospitals	TKM Clinics	Total	WM Hospitals	WM Clinics	TKM Hospitals	TKM Clinics	Total	WM Hospitals	WM Clinics	TKM Hospitals	Total	TKM Hospitals	TKM Clinics	WM Hospitals
**2013**	84,971	1,451	28,328	212	13,100	630,114	193,831	89,637	12,819	3,155	90,710	10,084	35,556	91	18,199	1,491	14,393	93
**2014**	86,629	1,474	28,883	231	13,423	660,099	194,424	86,688	14,329	3,593	92,927	10,342	36,475	136	18,767	1,446	14,798	95
**2015**	88,163	1,496	29,488	260	13,613	674,646	191,546	78,378	16,501	2,978	95,076	10,448	37,438	186	19,246	1,535	15,063	93
**2016**	89,919	1,514	30,292	282	13,868	692,500	191,683	75,338	17,979	2,920	97,713	10,774	38,770	223	19,737	1,596	15,392	99
**2017**	91,545	1,466	30,938	312	14,111	701,744	169,421	68,888	20,182	3,098	100,241	10,586	40,347	277	20,389	1,712	15,722	99
**2018**	93,184	1,465	31,718	307	14,295	707,349	165,302	62,863	20,038	3,368	102,471	10,730	41,845	287	20,759	1,782	15,992	101
**2019**	94,865	1,489	32,491	352	14,408	703,468	162,547	57,325	22,734	4,119	105,628	10,957	43,576	346	21,630	2,022	16,537	103
**2020**	96,742	1,515	33,115	410	14,464	716,292	165,107	55,418	26,494	5,227	107,976	11,072	44,781	434	22,038	2,265	16,748	124
**2021**	98,479	1,397	33,912	479	14,526	722,671	133,295	54,255	30,484	7,324	109,937	10,487	46,312	492	22,542	2,505	17,053	123
**2022**	100,396	1,398	34,958	546	14,549	724,212	132,262	53,350	34,378	7,933	112,321	10,450	48,584	559	22,807	2,708	17,159	119

TKM: Traditional Korean Medicine, WM: Western Medicine
