# Supplementary material for: Analysis of the utilization of traditional medicine in Korea over 10 years (2013–2022): A repeated cross-sectional study using national health insurance data
Source: PLoS One. 2025 Apr 8;20(4):e0321517. doi: 10.1371/journal.pone.0321517 (PMC11977961; doi:10.1371/journal.pone.0321517)
Supplement: S2 Table — (PDF) [file pone.0321517.s002.pdf]

S2 Table. Number of patients in Korea between 2013 and 2022

Year	WM Hospitals			WM Clinics			TKM Hospitals			TKM Clinics		
	Total	Inpatients	Outpatients	Total	Inpatients	Outpatients	Total	Inpatients	Outpatients	Total	Inpatients	Outpatients
**2013**	13,370	2,149	12,981	43,735	1,386	43,597	689	103	630	12,657	11	12,652
**2014**	13,458	2,308	13,019	42,891	1,342	42,765	685	107	623	12,811	12	12,806
**2015**	13,441	2,343	13,028	43,073	1,325	42,951	709	127	632	12,608	12	12,602
**2016**	13,787	2,381	13,391	43,470	1,296	43,361	728	140	641	12,499	12	12,494
**2017**	13,720	2,271	13,353	43,857	1,254	43,761	738	155	639	12,283	11	12,278
**2018**	14,054	2,356	13,683	44,214	1,236	44,130	743	154	645	12,067	11	12,062
**2019**	14,128	2,382	13,770	44,433	1,273	44,358	780	164	672	12,135	10	12,131
**2020**	12,801	2,097	12,474	42,822	1,217	42,749	758	158	651	11,124	10	11,119
**2021**	12,986	1,990	12,680	42,526	1,217	42,465	811	153	707	10,832	11	10,827
**2022**	15,338	2,003	15,049	45,912	1,210	45,860	849	164	735	10,736	13	10,731

Note. The unit of the values is in thousands. TKM: Traditional Korean Medicine, WM: Western Medicine
