# Supplementary material for: Analysis of the utilization of traditional medicine in Korea over 10 years (2013–2022): A repeated cross-sectional study using national health insurance data
Source: PLoS One. 2025 Apr 8;20(4):e0321517. doi: 10.1371/journal.pone.0321517 (PMC11977961; doi:10.1371/journal.pone.0321517)
Supplement: S3 Table — (PDF) [file pone.0321517.s003.pdf]

S3 Table. Number of claims in Korea between 2013 and 2022

Year	WM Hospitals			WM Clinics			TKM Hospitals			TKM Clinics		
	Total	Inpatients	Outpatients	Total	Inpatients	Outpatients	Total	Inpatients	Outpatients	Total	Inpatients	Outpatients
**2013**	62,846	3,222	59,624	515,039	1,798	513,241	3,422	219	3,203	97,704	17	97,687
**2014**	64,536	3,421	61,114	519,728	1,760	517,967	3,447	253	3,194	101,011	19	100,992
**2015**	65,357	3,497	61,859	514,167	1,752	512,415	3,471	331	3,139	99,141	20	99,121
**2016**	68,233	3,561	64,672	528,593	1,711	526,882	3,598	375	3,223	99,669	19	99,650
**2017**	67,708	3,372	64,336	534,432	1,674	532,759	3,684	426	3,258	99,817	18	99,798
**2018**	70,146	3,480	66,666	542,328	1,673	540,655	3,821	435	3,386	97,684	17	97,666
**2019**	71,556	3,543	68,013	552,099	1,753	550,346	3,997	474	3,523	100,561	16	100,545
**2020**	60,530	3,115	57,415	479,588	1,694	477,895	3,988	487	3,501	90,204	17	90,187
**2021**	60,482	2,945	57,537	470,941	1,726	469,215	4,459	489	3,970	89,168	18	89,150
**2022**	71,415	3,036	68,379	553,946	1,710	552,236	4,467	516	3,951	86,835	21	86,814

Note. The unit of the values is in thousands. TKM: Traditional Korean Medicine, WM: Western Medicine
