# Supplementary material for: Analysis of the utilization of traditional medicine in Korea over 10 years (2013–2022): A repeated cross-sectional study using national health insurance data
Source: PLoS One. 2025 Apr 8;20(4):e0321517. doi: 10.1371/journal.pone.0321517 (PMC11977961; doi:10.1371/journal.pone.0321517)
Supplement: S4 Table — (PDF) [file pone.0321517.s004.pdf]

S4 Table. Medical expenses reimbursed by national health insurance in Korea between 2013 and 2022

Year	WM Hospitals			WM Clinics			TKM Hospitals			TKM Clinics		
	Total	Inpatients	Outpatients	Total	Inpatients	Outpatients	Total	Inpatients	Outpatients	Total	Inpatients	Outpatients
**2013**	50,363	33,741	16,622	106,742	12,271	94,471	2,008	1,289	718	19,082	67	19,015
**2014**	51,931	34,484	17,446	113,134	12,235	100,899	2,196	1,452	744	20,528	76	20,452
**2015**	55,264	36,859	18,405	117,916	12,375	105,541	2,582	1,802	780	20,629	79	20,550
**2016**	58,786	38,843	19,943	126,477	12,460	114,018	2,981	2,124	857	21,224	78	21,146
**2017**	61,903	39,792	22,111	137,111	12,749	124,362	3,399	2,465	934	22,013	87	21,926
**2018**	68,519	44,001	24,518	151,291	13,352	137,939	3,583	2,597	986	23,613	92	23,521
**2019**	75,716	48,638	27,078	168,644	14,842	153,803	4,181	3,032	1,149	25,938	108	25,830
**2020**	77,535	50,946	26,589	170,342	15,828	154,514	4,840	3,691	1,149	24,660	156	24,504
**2021**	82,375	52,313	30,062	187,710	16,851	170,859	5,250	3,912	1,338	25,371	189	25,182
**2022**	90,799	53,470	37,329	231,199	17,140	214,059	5,739	4,307	1,432	25,793	245	25,548

Note. The unit of the values is in one hundred million Korean won. TKM: Traditional Korean Medicine, WM: Western Medicine
