# Supplementary material for: Analysis of the utilization of traditional medicine in Korea over 10 years (2013–2022): A repeated cross-sectional study using national health insurance data
Source: PLoS One. 2025 Apr 8;20(4):e0321517. doi: 10.1371/journal.pone.0321517 (PMC11977961; doi:10.1371/journal.pone.0321517)
Supplement: S5.1 Table — (PDF) [file pone.0321517.s005.pdf]

S5.1 Table. Claims for TKM examinations in Korea between 2013 and 2022

Year	Mederian Function			Pulse wave			Yangdorak			Personality			Dementia			Dizziness		
	Total	Inpatients	Outpatient	Total	Inpatients	Outpatient	Total	Inpatients	Outpatient	Total	Inpatients	Outpatient	Total	Inpatients	Outpatient	Total	Inpatients	Outpatient
**2013**	696,582	19,457	677,125	91,567	2,433	89,134	47,741	924	46,817	1,794	67	1,727	222	75	147	658	36	622
**2014**	665,320	20,321	644,999	94,331	3,027	91,304	40,153	919	39,234	2,301	72	2,229	245	91	154	212	24	188
**2015**	607,582	21,164	586,418	91,214	3,191	88,023	35,848	874	34,974	2,833	126	2,707	237	97	140	328	52	276
**2016**	575,551	19,599	555,952	82,676	2,801	79,875	32,984	643	32,341	3,966	110	3,856	601	77	524	356	86	270
**2017**	528,129	17,954	510,175	74,260	2,499	71,761	30,377	555	29,822	4,850	43	4,807	217	66	151	442	104	338
**2018**	500,495	14,973	485,522	74,575	2,720	71,855	28,014	329	27,685	5,781	56	5,725	233	55	178	497	76	421
**2019**	477,532	13,181	464,351	70,754	2,438	68,316	24,268	245	24,023	6,173	45	6,128	220	42	178	519	21	498
**2020**	423,445	14,251	409,194	69,022	1,896	67,126	20,476	208	20,268	6,625	51	6,574	213	62	151	373	16	357
**2021**	437,704	12,037	425,667	74,884	1,681	73,203	18,813	124	18,689	7,083	47	7,036	180	45	135	574	78	496
**2022**	445,827	9,263	436,564	82,951	1,820	81,131	18,823	96	18,727	6,992	61	6,931	208	45	163	631	66	565
