# Supplementary material for: Analysis of the utilization of traditional medicine in Korea over 10 years (2013–2022): A repeated cross-sectional study using national health insurance data
Source: PLoS One. 2025 Apr 8;20(4):e0321517. doi: 10.1371/journal.pone.0321517 (PMC11977961; doi:10.1371/journal.pone.0321517)
Supplement: S5.2 Table — (PDF) [file pone.0321517.s006.pdf]

S5.2 Table. Medical expenses for TKM examinations in Korea between 2013 and 2022

Year	Mederian Function			Pulse wave			Yangdorak			Personality			Dementia			Dizziness		
	Total	Inpatients	Outpatient	Total	Inpatients	Outpatient	Total	Inpatients	Outpatient	Total	Inpatients	Outpatient	Total	Inpatients	Outpatient	Total	Inpatients	Outpatient
**2013**	2,878,265	83,395	2,794,872	277,040	7,782	269,258	159,267	3,183	156,084	26,022	1,014	25,008	5,901	2,006	3,895	2,342	126	2,216
**2014**	2,821,487	89,548	2,731,939	292,015	9,848	282,167	137,418	3,226	134,192	33,976	1,123	32,852	6,630	2,481	4,150	772	91	682
**2015**	2,634,021	95,273	2,538,747	288,291	10,522	277,769	125,384	3,163	122,221	42,533	2,010	40,522	6,643	2,736	3,906	1,234	204	1,031
**2016**	2,550,027	90,282	2,459,745	267,347	9,428	257,919	117,890	2,406	115,484	60,512	1,791	58,721	16,403	2,185	14,218	1,388	355	1,032
**2017**	2,362,740	83,332	2,279,409	251,703	8,907	242,796	111,255	2,142	109,113	75,807	714	75,094	6,328	1,960	4,368	1,799	442	1,358
**2018**	2,143,752	66,549	2,077,203	282,877	10,879	271,997	104,835	1,287	103,548	93,469	957	92,511	6,968	1,684	5,284	2,192	347	1,844
**2019**	1,994,286	57,611	1,936,676	289,903	10,667	279,235	93,329	1,004	92,325	102,894	796	102,099	6,758	1,322	5,436	2,421	102	2,319
**2020**	1,719,086	60,266	1,658,820	303,973	8,900	295,072	80,467	841	79,627	113,747	930	112,817	6,749	2,030	4,718	1,839	82	1,756
**2021**	1,810,755	51,681	1,759,073	345,133	8,656	336,478	75,645	527	75,118	125,041	888	124,154	5,871	1,514	4,357	2,908	412	2,496
**2022**	1,894,971	40,980	1,853,989	425,738	12,479	413,261	77,784	429	77,356	127,270	1,214	126,055	6,987	1,572	5,414	3,315	363	2,953

Note. The unit of the values is in thousands Korean won.
