# Supplementary material for: Analysis of the utilization of traditional medicine in Korea over 10 years (2013–2022): A repeated cross-sectional study using national health insurance data
Source: PLoS One. 2025 Apr 8;20(4):e0321517. doi: 10.1371/journal.pone.0321517 (PMC11977961; doi:10.1371/journal.pone.0321517)
Supplement: S6.1 Table — (PDF) [file pone.0321517.s007.pdf]

S6.1 Table. Claims for TKM treatments in Korea between 2013 and 2022

Year	Acupuncture			Electroacpuncture			Cupping			Moxibustion			Heat-cold stimulation			Chuna			Others		
	Total	Inpatients	Outpatients	Total	Inpatients	Outpatients	Total	Inpatients	Outpatients	Total	Inpatients	Outpatients	Total	Inpatients	Outpatients	Total	Inpatients	Outpatients	Total	Inpatients	Outpatients
**2013**	204,409	18,505	185,904	20,390	1,790	18,600	43,837	3,307	40,530	27,112	4,398	22,714	46,273	1,485	44,788	-	-	-	1,615	128	1,488
**2014**	212,517	20,685	191,832	22,378	2,222	20,156	49,567	4,051	45,516	26,160	4,855	21,305	46,545	1,665	44,879	-	-	-	1,630	156	1,474
**2015**	211,600	22,839	188,761	25,235	2,660	22,575	48,872	4,941	43,932	26,299	5,331	20,968	45,045	1,912	43,132	-	-	-	1,493	199	1,295
**2016**	207,193	24,554	182,639	25,968	3,075	22,893	54,323	5,628	48,695	31,238	5,977	25,261	48,866	2,203	46,662	-	-	-	1,453	245	1,208
**2017**	211,480	25,512	185,968	28,860	3,412	25,447	51,061	6,132	44,929	31,268	6,394	24,874	47,526	2,164	45,362	-	-	-	1,763	405	1,358
**2018**	216,921	26,173	190,749	35,971	3,392	32,579	58,077	6,301	51,776	33,789	6,705	27,084	49,526	2,187	47,339	-	-	-	1,455	445	1,010
**2019**	225,053	26,089	198,964	37,217	3,469	33,748	64,275	6,374	57,901	32,700	6,948	25,752	51,671	2,359	49,313	3,174	156	3,018	1,390	441	949
**2020**	201,291	26,116	175,175	39,131	3,648	35,482	57,957	6,587	51,370	31,540	7,340	24,199	49,765	2,622	47,143	4,675	268	4,407	1,225	492	733
**2021**	202,133	26,631	175,502	40,675	3,745	36,930	55,199	6,926	48,272	34,032	7,965	26,067	50,723	2,811	47,912	4,856	266	4,589	1,202	516	687
**2022**	191,557	26,674	164,883	39,463	3,938	35,524	57,759	7,234	50,525	36,438	8,506	27,933	51,559	3,235	48,324	4,818	271	4,547	1,171	512	660

Note. The unit of the values is in thousands.
