# Supplementary material for: Analysis of the utilization of traditional medicine in Korea over 10 years (2013–2022): A repeated cross-sectional study using national health insurance data
Source: PLoS One. 2025 Apr 8;20(4):e0321517. doi: 10.1371/journal.pone.0321517 (PMC11977961; doi:10.1371/journal.pone.0321517)
Supplement: S6.2 Table — (PDF) [file pone.0321517.s008.pdf]

S6.2 Table. Medical expenses for TKM treatments in Korea between 2013 and 2022

Year	Acupuncture			Electroacpuncture			Cupping			Moxibustion			Heat-cold stimulation			Chuna			Others		
	Total	Inpatients	Outpatients	Total	Inpatients	Outpatients	Total	Inpatients	Outpatients	Total	Inpatients	Outpatients	Total	Inpatients	Outpatients	Total	Inpatients	Outpatients	Total	Inpatients	Outpatients
**2013**	810,759	77,963	732,796	88,615	7,876	80,739	216,185	13,842	202,343	77,878	12,160	65,718	78,738	2,484	76,254	-	-	-	4,847	376	4,471
**2014**	862,868	89,207	773,661	99,781	10,002	89,779	247,765	17,389	230,376	78,585	13,787	64,799	81,110	2,827	78,282	-	-	-	5,062	455	4,607
**2015**	872,911	100,472	772,440	114,955	12,274	102,681	254,027	22,109	231,919	81,361	15,522	65,839	79,448	3,322	76,126	-	-	-	4,846	617	4,228
**2016**	869,046	110,178	758,869	120,993	14,496	106,497	288,274	26,171	262,103	97,190	17,699	79,491	84,712	3,844	80,868	-	-	-	5,048	796	4,252
**2017**	905,258	115,927	789,332	133,251	15,974	117,277	287,625	29,236	258,388	101,295	19,637	81,658	88,472	4,194	84,279	-	-	-	6,487	1,477	4,881
**2018**	945,543	116,543	829,001	148,981	14,186	134,795	341,687	31,316	310,371	125,230	23,244	101,986	107,217	5,066	102,151	-	-	-	6,087	1,601	4,221
**2019**	989,538	115,416	874,122	143,191	13,461	129,731	391,409	33,194	358,215	132,460	26,026	106,434	124,501	6,028	118,473	85,216	4,903	55,174	6,199	1,559	4,374
**2020**	899,799	114,427	785,372	138,307	12,984	125,323	377,631	35,683	341,948	137,272	29,528	107,744	129,769	7,269	122,500	127,693	8,298	81,443	5,952	1,718	4,041
**2021**	928,143	118,241	809,901	146,483	13,502	132,982	381,349	38,431	342,917	151,459	32,463	118,996	135,581	8,051	127,530	136,772	8,353	87,726	6,328	1,831	4,313
**2022**	910,580	120,814	789,766	146,191	14,620	131,571	417,941	42,885	375,056	166,574	35,633	130,941	142,501	9,092	133,409	140,077	8,769	89,774	6,433	1,852	4,393

Note. The unit of the values is in millions Korean won.
